# The Relationship between Body Mass Index (BMI) and Depression According to the rs16139NPY Gene

**Published:** 2017-07

**Authors:** Atieh Dolatian, Seyed Masoud Arzaghi, Mostafa Qorbani, Hamideh Pishva

**Affiliations:** 1 Department of Cellular-Molecular Nutrition, School of Nutritional Sciences and Dietetics, Tehran University of Medical Sciences, Tehran, Iran.; 2 Elderly Health Research Center, Endocrinology and Metabolism Population Sciences Institute, Tehran University of Medical Sciences, Tehran, Iran.; 3 School of Medicine, Community Medicine Department, Alborz University of Medical Sciences, Karaj, Iran.

**Keywords:** *Depression*, *NPY Polymorphism*, *Obesity*, *Body Mass Index (BMI)*, *rs16139NPY Gene*

## Abstract

**Objective:** Obesity and depression are likely to interact mutually, which makes it unclear whether obesity causes depression or depression leads to obesity, and how the genotypes have a role in obesity and depression.

**Method:** This cross- sectional study was conducted on a sample of 400 individuals from the participants in the third phase of the comprehensive Iranian Multicenter Osteoporosis Study (IMOS). Anthropometric measurements and depression were assessed. PCR-RFLP was used to investigate the NPY polymorphism. Binary logistic regression model was employed to determine depression as the dependent factor and gene polymorphism.

**Results:** The frequency of NPY rs16139 was 6%. No significant association was found between NPY genotypes and depression (p >0.05). Furthermore, the results suggest that those with central obesity had an increased chance of developing depression (P = 0.02).

**Conclusion:** The frequency of NPY polymorphism was 6%. Our study could not find a correlation between rs16139 and depression.

Depression is a disorder characterized by loss of energy and interest, feelings of guilt, difficulty in concentration, loss of appetite and morbid thoughts of death and suicide. It is also associated with changes in activity level, cognitive abilities, speech, sleep, appetite and other biological rhythms (1). According to the World Health Organization, depression, after cardiovascular diseases, will be the second leading cause of burden of diseases by 2020. "Depression affects about 121 million people worldwide". Both depression and obesity impose great economic costs and disease burden worldwide, and the high prevalence of both might be an indicator of a relationship between them (2). There are observations suggesting that individuals with obesity suffer from depressive disorders. These findings have been supported by frequent reports of a positive association between obesity and depression.

Studies conducted by Sherwood et al. suggested that depression mediates the relationship between bulimia nervosa and weight loss (3). The Neuropeptide Y (NPY) polymorphism (rs16139) is a single nucleotide polymorphism (SNP) in which thymidine base substitutes to cytosine (T1128C). This substitution leads to impact on NPY secretion. People with this SNP (CC/CT genotype) have higher amount of NPY secretion (4). The role of hypothalamic NPY in energy balance has been studied well. Numerous studies showed that when this orexigenic neuropeptide chronically injects into the central nervous system, it causes an increased food intake and body weight in mice (5).

NPY is involved in some human diseases such as obesity, alcoholism, schizophrenia and depression, each of which might contribute to the development of psychotic behaviors (5). Future planning aimed at decreasing such diseases can positively reduce the costs of treatment, the costs imposed on the society, the burden of disease, and ultimately, mortality. Therefore, it is best to identify and control the neurobiological and pathological bases of these major disorders, which have remained unknown, rather than extensively investigating the various aspects of depressive disorders as a control theory for decades (6). Eventually, it can be concluded that obesity and depression are likely to interact mutually, which makes it unclear whether obesity causes depression or is it depression, which eventually leads to obesity; and how the genotypes involved in obesity and depression have a role in the depression of the obese. According to our investigation, to date, no research has been conducted to cover neuropeptide Y gene polymorphisms, obesity and depression in a single study in Iran. Perhaps this study could pave the way for a new approach to prevent obesity and depression, and hopefully, to cure them.

## Materials and Methods

This cross- sectional study was conducted on a sample of 400 individuals from the participants in the third phase of the comprehensive Iranian Multicenter Osteoporosis Study (IMOS) in Arak, Iran, which was a population-based study on 1,100 healthy adult male and female volunteers older than 20 years. Individuals weighing more than 120 kg., with acute or chronic liver or kidney failure, those with any type of cancer, as well as pregnant or lactating women, individuals who for whatever reason were hospitalized during the last two weeks prior to questioning, antidepressant consumers and those following a particular diet were excluded from the study.

Anthropometric measurements included weight, height, waist circumference, hip circumference and waist-to-hip ratio as previously described (7).

To determine the depressive disorder, we used the Structured Clinical Interview (SCID). The SCID is a semi-structured interview for making the major DSM-IV diagnoses. Finally, the severity of depressive disorder was determined, using the standardized Beck Depression Inventory (BDI II).

Genomic DNA was extracted from the whole blood using Gene All kits (ExgeneTM cell SV kit); 48 and 190 bp DNA fragment, containing the thymidine to cytosine substitution in the NPY gene (4), was genotyped by PCR-RFLP procedure. The forward and reverse primers used to detect the exon 2 of the NPY gene were 5’-CCCGTCCGTTGAGCCTTCTG-3’ and 5’-CGGTCCCGCGGTCCC-3’, respectively (8). PCR conditions included an initial denaturation at 95 °C for two minutes, followed by 35 cycles of 30 seconds at 95 °C (denaturation), 30 seconds at 62 °C (annealing) and 50s at 74 °C (extension). Final extension was five minutes at 72 °C. PCR products were digested as follows: 7µl of the product was incubated with 0.3 µl of the BsiE1 and 2 µl of 10× restriction G-buffer (in total 20 µl reaction), overnight at 37 °C. To investigate the presence of polymorphism, the digested samples were electrophoresed on a 2% agarose gel and stained with green viewer.

SPSS Version 16 was used to analyze the produced data. The quantitative data are presented as Mean ± SD. The genotype distribution and allele frequencies were analyzed, using chi-square. T-test was used for the quantitative data with normal distribution. Binary logistic regression model was employed to determine depression as the dependent factor and gene polymorphism, age and gender as covariables in the model. Because only a few subjects with CC were found among the participants, they were pooled with CT subjects and analyses were carried out. OR and confidence interval were calculated and a p value <0.05 was considered significant.

## Results

A total of 400 people entered the study. The mean age of the participants was 43.84 ± 12.61. Moreover, 45.5% and 54.5% of the participants were male and female, respectively. No significant difference was observed for both depression and NPY polymorphism (between the two BMI category groups). Likewise, no significant difference was observed between anthropometric measurements and depression in groups. However, there was a significant difference between waist circumferences and BMI with BMI≥25 group. Table 2 showed that depressed persons in BMI ≥ 25 had higher waist circumference than non-depressed persons.

The anthropometric measurements of the participants were analyzed between NPY genotypes. The results indicated no significant difference between the variables and genotypes (Table 3).

Multiple logistic regression results did not find any significant association between NPY genotypes and depression after and before adjusting the risk factors (age and sex as covariables) (Table 4).

 Furthermore, the multiple logistic regression suggest that central obesity (waist to hip ratio >1) had an increased chance of developing depression by 56% before and after adjustment of the risk factors (Table 4).

**Table1:** The Association of Anthropometric Measurements with Depression in different groups

Variables	**BMI < 25 n = 200**			**P**	**BMI ≥ 25 n = 200**		**P**	**Total n = 400**		**P**
Mean(SD)	Non depression	Depression			Non depression	Depression		Non depression	Depression	
Weight (kg)	69.5 (9.12)	63.02 (8.39)	0.3		77.9 (9.78)	80.26 (10.2)	0.06	72.66 (11.34)	70.46 (12.58)	0.1
BMI (kg/m^2^)	22.83 (1.61)	22.73 (1.61)	0.7		28.71 (3.20)	30.57 (3.84)	<0.01*	26.62 (3.93)	26.12 (4.8)	0.3
Hip Circumference (cm)	95.99 (9.74)	95.96 (9.29)	0.9		1.05 (1.12)	1.08 (1.39)	0.09	1.01 (1.22)	1.01 (1.30)	0. 7
Waist circumference (cm)	81.06 (1.37)	78.82 (9.21)	0. 2		91.26 (1.29)	96.13 (1.56)	0.03*	87.61 (1.41)	86.29 (1.5)	0. 4
WHR	0.84 (0.12)	0.82 (0.09)	0. 2		0.87 (0.09)	0.88 (0.09)	0.3	0.86 (0.1)	0.85 (0.09)	0.3

* Significant Difference by Independent T- Test

**Table2:** The Association of NPY Variant rs16139 with Anthropometric Measurements in different groups

**Mean (SD)**	**BMI < 25** **N = 200**			**BMI ≥ 25** **N = 200**			**Total** **N = 400**		
**Variables**	**Genotype**		**P** *	**Genotype**		**P** *	**Genotype**		**P** *
	CT/ CC	TT		CC/CT	TT		CC/ CT	TT	
Weight (kg)	73.34 (11.38)	71.99 (11.7)	0.6	76.65 (12.22)	77.88 (9.80)	0.6	66.16 (4.21)	63.86 (9.04)	0.5
BMI Kg/m^2^	28.18 (6.10)	26.39 (4.05)	0.07	30.32 (6.32)	29.01 (3.15)	0.4	23.54 (0.83)	22.77 (1.68)	0.07
Waist circumference (Cm)	87.68 (16.59)	87.23 (14.24)	0.8	93.00 (14.43)	92.19 (13.46)	0.8	76.16 (16.08)	80.47 (12.09)	0.4
WHR	0.83 (0.08)	0.86 (0.1)	0.3	0.85 (0.08)	0.87 (0.09)	0.3	0.79 (0.05)	0.83 (0.11)	0.4

* Independent T-Test

**Table3:** The Association between rs16139 and Depression in the Logistic Regression Model in different group

**Groups**	**Genotype**	**Depression**					
		OR	95%CIs	P	OR^a^	95%CIs^a^	P^a^
Total	CT,CC/TT	0.9	0.333, 2.708	0.9	0.882	0.307, 2.534	0.815
BMI<25	CT,CC/TT	1.7	0.333, 8.763	0.5	1.784	0.333, 9.545	0.4
BMI≥25	CT,CC/TT	0.7	0.152, 3.326	0.6	0.749	0.156, 3.583	0.7

a Adjusted for Age and Sex

**Table4:** The Association between Central Obesity and Depression in the Logistic Regression Model

**Group**	**Anthropometric variable**	**Depression**					
		OR	95%Cis	P	OR^a^	95%CIs^a^	P^a^
Total	Central obesity	0.586	0.307, 0.928	0.023	0.567	0.350, 0.919	0.02
BMI<25	Central obesity	0.546	0.282, 1.056	0.07	0.567	0.350, 1.093	0.08
BMI≥25	Central obesity	0.680	1.256, 1.739	0.6	0.561	0.283, 1.307	0.2

a Adjusted for Age and Sex

## Discussion

The frequency of NPY rs16139 is distinct among different populations with varied genetic backgrounds. Our results revealed that the frequency of NPY polymorphism was 6%. According to Ding et al., the prevalence of this polymorphism is 6-15% among the Caucasians and it is relatively rare in Asians (Korea and Japan) (9). However, Yanggjun reported the proportion to be standing at about 1% among Han Chinese (6). In a study by Massoudi in Mashhad, Iran, on CAD patients, the polymorphism prevalence was 5.9% (4). This is close to Massoudi’s results and the Caucasian population. The relationship between waist circumference, and abdominal obesity as components of the metabolic syndrome on the one hand and mental illness on the other hand, has already been investigated (10). Studies on 35-55-year old adults in London (11), 25-84-year old adults in Australia (12), and 20-70 year old men in Japan (13), all indicated a significant relationship between waist circumference and depression. Data analysis revealed that a significant correlation was observed between waist circumference, BMI and depression among the overweight and obese group, highlighting the concept of a significant relationship between obesity and depression. The results of studies by Dunbar in 2008 (12), Akbaraly in 2009 (11), and Zhao in 2011 (10) are consistent with ours. Although age is an effective factor in depression, no significant link was found between age and depression in our study. Takeuchi stated waist circumference in patients with metabolic syndrome as the most important factor in the relationship between depression and obesity (13). Dunbar found that abdominal obesity leads to activation of inflammatory pathways and mediators in the long term and cause depression (12). In view of the aforementioned, it was finally concluded that obesity is a possible cause of depression in the general population. The inhibition of NPY secretion in the brain has been reported in animal models of depression, while antidepressant treatments were found to increased synthesis of NPY in the brain. In this regard, injection of NPY into some parts of the brain develops antidepressant effects. Observations which support the role of NPY in human depression include depressed patients and suicide attempters withlow levels of NPY in plasma and cerebrospinal fluid (5). The results of our study did not show a correlation between rs16139 and depression although Yanggjun (6) and Sjoholm (14) found a significant association between depression and Leu7 Pro polymorphism, and so their findings vary from ours in this respect. Nevertheless, Domschkeet al. (15) compared this polymorphism with other alleles of NPY and found that this particular rs16139 is not related to the perceived effects of treatment and prognosis of patients with depression. The participants in this study were divided into two groups according to their BMI and then the prevalence of depression, polymorphism, and the concerned relationships were assessed. As a result, to support a cause and effect relationship, comparison of polymorphism between the depressed and non-depressed participants seems necessary. Another contributing factor to be mentioned is the varied etiology of depression in different populations. While other studies that found a relationship between NPY and depression measured its levels in plasma or CSF of the depressed patients or animal models (4), in this study, NPY levels were not measured in plasma or cerebrospinal fluid of the participants. The effects of NPY on depression are still not clearly known. Thus, further studies to explore the relationship between the corresponding causes and effects are certainly required. In this study, no significant difference was found between the anthropometric indices such as weight, BMI, waist circumference, waist-to-hip ratio, and NPY polymorphism. Kakko in 2011(16), Ukkola (17) and Karvonen (8) in 2007 were not able to find a significant difference between BMI and this polymorphism. Kakko had measured waist circumference in the subjects, but still could not find a significant difference. Van Rossum observed the association of this polymorphism with obesity only in the men, and suggested that the effect of this polymorphism may be initiated in younger ages (18). However, Massoudi found a significant correlation between BMI, weight and this polymorphism (4). Mattevi (19) reported a significant relationship only between BMI and a certain group of women (pre-menopause); and this may have been due to the low prevalence of this polymorphism, differences in the characteristics of the subjects, and presence of factors reducing the polymorphism-induced defect, such as lifestyle or other environmental factors.

## Limitations

In this study, NPY levels were not measured in plasma or cerebrospinal fluid of the participants. 

## Conclusion

A relationship was found between obesity and depression; however, NPY polymorphism did not affect depression or obesity.
